# Organizational Culture Shapes the Adoption and Incorporation of Simulation into Nursing Curricula: A Grounded Theory Study

**DOI:** 10.1155/2014/197591

**Published:** 2014-04-10

**Authors:** Karyn Taplay, Susan M. Jack, Pamela Baxter, Kevin Eva, Lynn Martin

**Affiliations:** ^1^Department of Nursing, Brock University, 500 Glenridge Dr., St. Catharines, ON, Canada L2S 3A1; ^2^School of Nursing, McMaster University, 1280 Main Street West, Hamilton, ON, Canada L8S 4K1; ^3^Centre for Health Education Scholarship, University of British Columbia, 950 West 10th Avenue, Vancouver, BC, Canada V5Z 1M9

## Abstract

*Purpose*. To create a substantive mid-range theory explaining how the organizational cultures of undergraduate nursing programs shape the adoption and incorporation of mid-to high-level technical fidelity simulators as a teaching strategy within curricula. *Method*. A constructivist grounded theory was used to guide this study which was conducted in Ontario, Canada, during 2011-12. Semistructured interviews (*n* = 43) with participants that included nursing administrators, nursing faculty, and simulation leaders across multiple programs (*n* = 13) informed this study. Additionally, key documents (*n* = 67) were reviewed. Purposeful and theoretical sampling was used and data were collected and analyzed simultaneously. Data were compared among and between sites. *Findings*. The organizational elements that shape simulation in nursing (OESSN) model depicts five key organizational factors at the nursing program level that shaped the adoption and incorporation of simulation: (1) leaders working in tandem, (2) information exchange, (3) physical locale, (4) shared motivators, and (5) scaffolding to manage change. *Conclusions*. The OESSN model provides an explanation of the organizational factors that contributed to the adoption and incorporation of simulation into nursing curricula. Nursing programs that use the OESSN model may experience a more rapid or broad uptake of simulation when organizational factors that impact adoption and incorporation are considered and planned for.

## 1. Introduction

Organizational culture is defined as the ways in which people know and understand the values and beliefs of a specific group of people or an institution [1]. These values and beliefs are established over time, are considered valid, and are taught to new members who enter into the culture [1, 2]. Organizational beliefs and values are guiding principles that influence the development of individuals' attitudes towards the organization and how individuals within that culture make decisions or invest their time [3]. Academic nursing programs are situated within challenging and complex organizational cultures [4] characterized by a combination of the traditional research, teaching, and service requirements of academia coupled with professional practice requirements [5]. Added to the complex culture of academic nursing is the integration of new technology in conjunction with a demand for diverse teaching strategies [5]. Simulation, specifically using mid- to high-fidelity equipment, which closely mimics real life experience without the real life risks, [6] is considered new technology. Although simulation has been used in nursing education programs since the early nineteen hundreds [7, 8], advances in the technological capabilities and the types of simulator equipment available today reinforce the notion of simulation being viewed by nursing faculty as a new teaching strategy. Additionally, there have been inconsistent levels of adoption and incorporation of simulation among individual nurse educators and across nursing programs [9]. Individual factors such as faculty members' attitudes and perceptions of simulation have been studied [10–12], but there is a gap in the literature related to how organizational culture shapes and contributes to attitudes, perceptions, and behaviors that impact the adoption and incorporation of simulation into nursing curricula.

Akhtar-Danesh and colleagues [9] conducted a q-methodology study in Canada of nursing faculty members' perceptions related to the adoption and integration of simulation. Benefits included aligning with students' positive perceptions about the use of simulation as a valuable teaching strategy, but challenges were identified about the usefulness and practicality of simulation in undergraduate education. Other researchers have reported that having the time to learn about and use simulation as well as having the resources to coordinate simulation-based education are the most significant barriers related to adoption and incorporation [12–14].

In the nursing education literature, identification of the individual perceptions, values, and concerns that influence the uptake of simulation have been well documented. While there has been some preliminary work to identify organizational influences on this issue such as the lack of faculty time and resources to implement this new technology [12–14], what has been consistently lacking is an examination of the organizational culture that leads to the development of attitudes, values, beliefs, and perceptions among nursing faculty as they respond to change or adopt an innovation. There is more to integrating simulation into nursing curricula than faculty buy-in. The choices people make and the activities they invest in are not always guided by personal choice but by the culture of the organization. Organizational factors have been shown to significantly impact organizational change [15]. Therefore, it is important to examine how organizational factors impact the adoption and incorporation of simulation as a teaching strategy as it is becoming more prevalent in nursing education and as institutions look for ways to facilitate this change.

Schein's [15] theory of organizational culture was used to sensitize the researcher about concepts of organizational culture. This theory provided both a clear definition of organizational culture and distinguishable levels of culture that should be considered [16]. Although grounded theory research is not typically conducted using an existing theoretical framework, Schein's model was used to ensure exploration of core constructs associated with organizational culture. Schein defines organizational culture as the ways of knowing within a specific group that have been established over time and have been shown to be effective in managing problems. They are considered valid, taught formally and informally to new members, and include both explicit and tacit knowledge [1]. Schein suggests there are three major tenets to consider when analyzing organizational culture: artifacts, espoused beliefs or values, and basic assumptions, each representing different levels of culture. Artifacts are predominantly the observed characteristics of organizational culture such as building design and structure and dress norms; these represent the most superficial level of organizational culture. Schein suggests that the best way to understand organizational culture is to examine it at deeper levels.

Beliefs and values are considered legitimate and significant guiding principles of an institution [1, 2]. They are considered ethical rules or philosophies of practice. They are the goals, aspirations, and ideologies that signify what is most important to an organization or for what the organization is ultimately striving. Beliefs and values are often represented in public statements such as mission and vision statements [10]. At the deepest level of Schein's model are the basic assumptions which are thought to guide actions, contribute to attitudes and behaviours, and impact thoughts and feelings. They are often at an “unconscious or taken for granted” [10] level and need to be uncovered to understand the influence they hold.

Nursing programs in Ontario, Canada, provided ideal circumstances in which to consider this issue. In 2004, the Ontario Ministry of Health and Long-Term Care demonstrated a commitment to nursing education through a $20 million investment for the purchase of simulation equipment for undergraduate nursing programs [17]. Monies were distributed to all 34 programs, ranging from $196,300 to $706,400, with the average institution receiving $500,000. Simulation equipment was the only allowable expense and nursing programs were expected to secure funds to ensure the continued operation and use of the equipment [18]. Charged with this expectation, nursing programs had to undergo a process of adopting and incorporating simulation into the curricula. So many nursing programs dealing with this initiative at the same time provided a good opportunity to examine the organizational elements that impacted this change. As the focus of the funding was mid- to high-fidelity simulation equipment, virtual simulations and standardized patients were excluded from this study.

## 2. Methods

### 2.1. Design

The procedures and principles of constructivist grounded theory [19] guided this research study. Charmaz's approach served to answer the research question: how do the organizational cultures of Ontario undergraduate programs of nursing shape the adoption and incorporation of simulation as a teaching and learning strategy within the curricula?

### 2.2. Research Setting

This study was conducted in Ontario, Canada. Since 2005, a baccalaureate degree has been the entry to practice requirement [20]. As a result, colleges in Ontario have worked in partnership with universities to offer collaborative nursing programs where students can obtain a baccalaureate degree. Collaborative programs are offered as articulated or integrated models. In articulated collaborations, the first two years of the nursing program are offered at the college and the second two years are offered at the university. In integrated collaborations, the delivery of the program happens at all of the sites, and the faculty members from both the college and university are involved throughout each year of the program. Nursing programs were selected based on the following criteria: (a) listed on the College of Nurses of Ontario web site [20] as an accredited program, (b) offered a four-year baccalaureate degree in English either on its own or in collaboration with another educational institution located within Ontario.

### 2.3. Sampling

Nursing programs that met the inclusion criteria were separated into geographical regions and then selected by stratified randomization to increase variability and reduce bias. Maximum variation among institutions was achieved by including both types of collaborations. Theoretical sampling as a form of purposeful sampling was used to ensure the core constructs and the deepest levels of organizational culture were examined [15]. The examination of artifacts was excluded from this study since there was an absence of equal opportunities to examine the artifacts at all of the sites. Knowledge about the beliefs and values was obtained by studying guiding documents such as the mission, vision, or philosophy statements at the level of the institution, the faculty, and the nursing program. Sampling to investigate basic assumptions, the deepest level of organizational culture, occurred at the level of the nursing program through in-depth semistructured interviews with participants. Participants from multiple roles within the program included nursing administrators, nursing faculty members, and key simulation leaders, thereby facilitating maximum variation sampling. All participants were contacted by publicly available email addresses. Snowball sampling was used to identify additional participants and documents [21, 22]. Theoretical sampling was used as data collection progressed and categories and themes started to emerge. This continued until data were sufficient to provide detailed descriptions of the experience from the participants' perspective. That is, sampling continued until theoretical sufficiency was obtained [19, 23].

### 2.4. Data Collection

Multiple data types and sources were collected as part of this study. This included up to two in-depth semistructured interviews with nursing administrators, nursing faculty members, and simulation leaders. First interviews lasted approximately 60–75 minutes and explored organizational factors involved in the processes of adoption and incorporation of simulation. Second interviews, lasting 30–60 minutes, were conducted to further develop the categories emerging from the data. To gain a rich description of the organizational culture, key documents were collected. These documents were compiled and sent to participants in advance of the interview. During the interviews, participants were asked if and how the documents shaped the adoption and incorporation of simulation into the curricula. Participants were also asked if any other documents contributed to this process and to share those documents when possible. All but one interview was completed by the primary investigator KT. NVivo 9 qualitative software was used to store, organize, and manage all data [24].

### 2.5. Data Analysis

A characteristic of grounded theory research is concurrent data collection and analysis [19]. Interviews were transcribed verbatim; then, data were analyzed line-by-line and incident-by-incident resulting in multiple codes, many of which were unique terms used by the participants (*in vivo* codes). Coding of data followed process outlined by Charmaz (2006) [19]. The initial codes moved to focused codes that served to develop analytical categories for further examination and exploration. As concurrent data collection and analysis continued, tentative categories started to emerge. Once the tentative categories were further explored and developed, they were raised to conceptual categories which are more abstract and led to the nascent structure of the theory. Documents were analyzed for key concepts and compared to interviews to see if the concepts were captured implicitly, explicitly, or at all. During data analysis a seven-phase process of adoption and incorporation emerged which resulted in the differentiation of high-, mid-, and low-uptake sites [25]. The constant comparative method was used to explore how organizational culture impacted or shaped the process [26]. To facilitate this, creating memos and theoretical sampling were used to capture thoughts, define codes, illuminate properties, make connections, and explore emerging themes. During this process the researcher looked for confirming and disconfirming examples to challenge the developing theoretical structure. This allowed for further exploration and expansion of emerging categories until theoretical saturation was reached [19]. Once categories were condensed the structure of the theory emerged.

### 2.6. Ethics

Approval was obtained through two university-based research ethics boards. Verbal and written consents were obtained from all participants. Participation was voluntary and participants could withdraw from the study at any time or choose not to answer questions. Identifying information was removed from all documents to maintain anonymity and confidentiality.

## 3. Results

### 3.1. Characteristics of the Participants, the Documents and the Institutions

Representatives of thirteen nursing programs across the province of Ontario, including seven universities and six colleges, participated in this study. Of these sites seven were identified as high-uptake, five as mid-uptake, and one as low-uptake. 55.5% of participants worked in the high-uptake sites, 40.7% at mid-uptake sites, and 3.7% at the low-uptake site. All four geographical regions of the province were represented. 43 semistructured interviews were conducted with 27 participants. The participants were all female and all registered nurses. Their roles included simulation leaders (*n* = 6), nursing administrators (*n* = 5), nursing faculty (*n* = 12), and those serving in a combined role (*n* = 4). All had a baccalaureate degree with the majority having a master's degree (85.1%); 14.8% had a PhD. The majority (75%) were between the ages of 41 and 60 years. See Table 1 for a detailed summary of the demographics.

A total of 67 documents were collected and analyzed and each organization contributed documents. Refer to Table 2 for a summary of the type of documents and the level of the institution they represent.

### 3.2. Organizational Elements That Shape Simulation in Nursing (OESSN) Model

**Figure 1:**
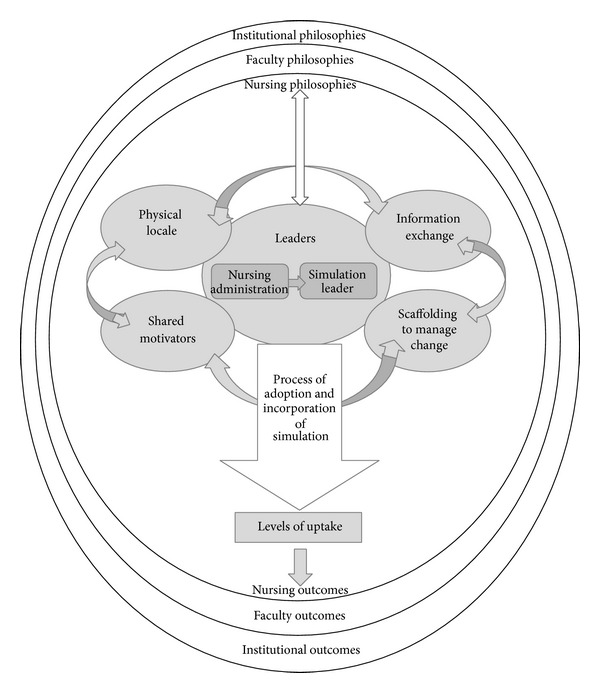
The organizational elements that shape simulation in nursing.

To explain how educational organizational cultures influence the adoption and incorporation of simulation, a theoretical model identified as the organizational elements that shape simulation in nursing (OESSN) model, Figure 1, was developed. A brief description of the full model will be followed by an in-depth explanation of each of the key organizational components. The model displays concentric circles that represent the different levels of the organization that impact the adoption and incorporation of simulation into nursing curricula. The outermost circle represents the institution. The next circle represents the Faculty of Health (or otherwise named) and the innermost circle represents the nursing program. Each of these three levels has within it values and beliefs, which are termed guiding philosophies, and contributes to the organizational culture of the nursing program. Within the level of the nursing program, five key elements of the organizational culture emerged. They represent what Schein [15] asserts are basic assumptions in that they influenced perceptions and attitudes and impacted behaviours and actions related to the adoption and incorporation of simulation. They are (a) nursing leaders, (b) information exchange, (c) physical locale, (d) shared motivators, and (e) scaffolding to manage change.

The central organizational element among the five was the nursing leaders who were typically nursing administration and the key simulation person (simulation leader). Within the model, there is an arrow from the nursing administrator to simulation leader indicating the distribution of power required in the creation of a new role to manage the simulation initiative.

The five key organizational elements impacted the seven-phase process of adoption and incorporation, represented in the model by the large downward arrow. The phases include (a) securing resources, (b) nursing leaders working in tandem, (c) getting it out of the box, (d) learning about simulation and its potential for teaching, (e) finding a fit, (f) trialing the equipment, and (g) integrating into the curriculum [25].

The seven-phase process leads directly to the levels of uptake which occurred across a continuum. High-uptake was defined as nursing programs that moved through all of the seven phases of the process and successfully integrated simulation into all levels of the curricula in which nursing content is taught.

Institutions that met some, but not all, phases of the process were classified as mid-uptake and institutions that were not able to progress through any of the phases were classified as low-uptake [25]. Institutions that were classified as high-uptake experienced more outcomes than the other sites and the outcomes had the potential to cross all levels of the organization. The remainder of this paper will provide in-depth explanations of the organizational elements that shape the adoption and incorporation of simulation into nursing curricula and will, at times, be discussed in relation to the level of uptake. The quotes that are used represent the voices of the participants. They were selected amongst all of the data to illustrate or provide an example of the broader context being described.

### 3.3. Guiding Philosophies

Before discussing each of the organizational components in detail, it is useful to discuss the relationship between the level of uptake and the guiding philosophies. The guiding philosophies, in the form of mission, vision, value, or philosophy statements, impacted on nursing programs' capacity to adopt and incorporate change. They are rooted within the faculty and institutional levels of the organization. Each level has beliefs and values that stem from the overarching organizational culture. These beliefs and values influence actions and decision making when managing change.

The explicit knowledge and use of the organizational guiding philosophies varied considerably across the uptake continuum. Many of the high-uptake sites consciously used the mission and vision statements of the institution, faculty, and nursing program as a method to make decisions and situate simulation within the curricula. When asked about adopting and incorporating simulation, one simulation leader indicated this by stating: “[simulation] relates to the mission, it's innovative … being involved in things that are innovative is part of our mission (006).” Specifically, the terms innovative, research focused, experiential learning, and quality of students' experiences, as well as the institutional teaching and research components facilitated decision making with regard to simulation.

This conscious explicit use of the guiding philosophies was not unidirectional. Nursing leaders used the guiding philosophies as a strategy for change but also as a tactic to acquire necessary resources. This is represented by the two-way arrow in the OESSN model between the leaders and the concentric circles representing the guiding philosophies. One nursing simulation leader reinforced this when stating:

“So anything that we do … has to somehow relate—if we're asking for resources, financial or human, it has to relate to the mission, vision, or values in order to be accepted. So any proposal that I bring forward, I ensure that it relates to those statements and I include rationale as to how this meets our mission, vision, or values (002).”

In the mid-uptake sites the impact of guiding assumptions was much more implicit. Participants used language such as “cutting edge,” “engaging students in learning,” and “evaluating simulation” during the interviews that mirrored or resembled the key concepts of the institutional, faculty, or nursing program guiding documents. When questioned directly about the influence these documents had on the adoption and incorporation of simulation, participants needed to refer back to the guiding documents in order to make connections. One simulation leader emphasized this stating: “I'm sitting here looking at the two mission statements … I see the words innovation and developing and disseminating knowledge … I see that as related [to simulation] (020).” On the other end of the spectrum is the low-uptake site. When questioned about how the guiding philosophies shape the adoption and incorporation of simulation a nursing a faculty member responded saying “there is nothing in our statements specific to simulation (027).” The findings show that it is was not necessarily the specific language of the underlying beliefs and values but the way in which leaders and nursing faculty members chose to use them that shaped the adoption and incorporation of simulation.

### 3.4. Leaders

With an initiative as extensive as this one, multiple resources and people are needed. Many nursing administrators had this insight realizing they would not be able to do it all themselves, so simulation leaders were appointed or volunteered. This was the beginning of the shared leadership that has shaped the adoption and incorporation of simulation in nursing curricula. This was noted by one simulation leader as follows: “our former chair … was instrumental in giving me …. the green light to go ahead and take the lead on this (001).” This shared leadership became the driving organizational force that was required to implement simulation as a teaching and learning strategy in nursing curricula. The leaders consisted primarily of nursing administration and the simulation leader. They were the key organizational element and served as the axis on which the other essential organizational elements interacted. The leaders did not act as individual leaders working on separate tasks but shared strategic leadership working in tandem toward a common goal. One nursing administrator highlighted this saying

“In any successful venture one person can't do it all … it was a joint effort … everybody brings their own unique knowledge and skill to the table. The ideas for the strategic plan were mine but without [the simulation leader] it wouldn't have gone anywhere and still would be an idea (012).”

The nursing leader's role is multifaceted and required the leader to engage multiple strategies to facilitate the simulation initiative. These strategies included negotiating, navigating, and networking. Negotiating involves working with nursing faculty members within the nursing program as well as with groups or individuals at the level of the faculty and the institution to exchange ideas and secure necessary resources. Navigating includes mapping out a certain course or plan for simulation within the nursing curriculum. Networking consists of generating a support system within the institution or among community members who have similar interests in simulation or can assist with the navigating process [27]. Much of what drove the leaders to support the simulation initiative were the overarching guiding philosophies. These statements articulate what is significant or important to the institution, faculty, and nursing program and, in turn, imply value. For example, institutions that promoted innovation within their mission or vision statements had nursing leaders that used that value statement as a rationale to secure resources and promote the implementation of simulation into the curriculum because it ultimately aligned with and supported the mission of the institution. Another driving factor among nursing leaders was the expectation of use and the shared motivators that are discussed in relation to the four remaining organizational elements.

### 3.5. Information Exchange

Information about simulation, its potential for enhancing the teaching and educational experience, and information about the additional resource requirements needed to support it had to be shared between nursing leaders, nursing faculty members, collaborative partners, and among different levels within the institution. The exchange of information about simulation was required at the onset of adoption and incorporation to get it started, but ongoing communication was also required to maintain simulation as part of the nursing curriculum. Information was communicated in a variety of ways and was significantly influenced by the nursing leaders who used formal and informal as well as written and spoken communication. One formal strategy used by several high-uptake sites was student evaluations of simulations. These evaluations included student feedback about the satisfaction with, or perceptions of, simulation as a teaching/learning strategy. The summaries of these evaluations were presented at formal meetings as a way to facilitate the formal exchange of information among individuals or groups that were using simulation and those who were considering using it or uncertain about using it. The nursing leaders who facilitated this type of formal communication placed value on simulation and provided ongoing opportunities for simulation to be considered as a teaching/learning opportunity. One nursing administrator explained this saying

“We've built in evaluation all along … then twice a year we invite the sim coordinator to do a presentation to faculty about the events and activities that have occurred … and I think what that does is keeping the thinking process alive and helping faculty members anticipate what they might consider in their own courses (025).”

All sites used some formal information exchange strategies such as introducing simulation at faculty or curricula meetings or learning about simulation at conferences. However, the majority of low and mid-uptake sites used fewer formal exchanges of information and used them less frequently than the high-uptake sites. This was articulated by one faculty member stating “we don't have a formal process; it's more informal discussions (023).” This informal exchange of information was also more prevalent and diverse in the high-uptake sites and occurred in a variety of ways. Predominantly, it was centered on one-on-one or small group discussions. Also included were email updates about simulation, discussions about research findings, or displays of the findings. One simulation leader maintained this saying “our manager would just send something out via email and it was by word-of-mouth (021).” Informal information exchanges strategies often served to promote interest and generate ideas about simulation. Multiple and diverse ways of communication facilitated movement through the seven-phase process of adoption and incorporation. Exchange of information was needed for all of the phases but was crucial in the phases of learning about simulation and its potential for teaching, finding and fit, and securing additional resources. The transfer of information about simulation between all levels of the organization was needed to secure a place for it in the curriculum.

Information exchange is not only influenced by the communication strategies of the leaders, but also shaped by the physical environment. One faculty member recognized this stating “our offices were across the hall from each other so we were always bouncing ideas off of each other (024).” Having people work in close proximity allowed for spontaneous conversations and sharing of ideas related to simulation. This concept of physical environment leads to the subsequent organizational element, physical locale that shaped the adoption and incorporation of simulation into nursing curricula.

### 3.6. Physical Locale

A significant amount of space is required to store, maintain, and utilize simulation equipment. When the initial request for funding was advertised, institutions were required to show the appropriate allocation of space or a renovation plan. This resulted in many nursing administrators having to fight for space and many simulation leaders having to deal with the space provided. A multitude of solutions regarding the physical location of the simulation lab ensued. Space was rented that was located away from the nursing program on another campus, in a different part of the city, or on another campus. Many nursing labs were renovated to accommodate the simulation equipment. Many participants claimed that the physical environment of the lab shaped the adoption and incorporation of simulation into nursing curricula. Often the remote physical location of the lab was isolating for many simulation leaders and created disconnects between people making it difficult to share or generate ideas. One simulation leader stated “The labs are completely separate from the nursing program and that also is a little bit tricky … because sometimes people don't want to come over here (007).” The isolated location of many simulation labs leads to feelings of frustration and the perception that simulation as a teaching strategy was undervalued or unsupported by the institution. This impeded the ability of some institutions to move successfully through all of the phases in the process.

Despite the isolation and disconnection that occurred in many new spaces, institutions which renovated spaces also had their own unique challenges. Renovated spaces were often described as inadequate or inefficient because of lack of knowledge about simulation equipment during the planning of the renovations. One simulation leader summed this up stating “The way our simulation area has been set up is not the best layout. Our control room is actually down the hall … it's not even connected to the space that we're in … it wasn't a well thought out design (027).” Renovations completed during the early stages of this initiative before stakeholders had a full understanding of the complexity of the equipment often resulted in frustration among simulation leaders and other users and ineffective use of the equipment.

The physical locale proved to be a significant organizational element that shaped the adoption and incorporation of simulation in shared nursing curricula being offered among collaborative partners. Within the context of this study, nursing programs are offered in collaboration with college and university partners; typically collaborations comprised two or three institutions. There is a physical distance between the institutions as most collaborative partners are not housed in the same location. Despite sharing curricula, the physical locale of the lab in separate physical environments provided a greater organizational influence than the collaborative partnership. One faculty summed this up saying “although we are a collaborative program, we are not really collaborative with regard to sim. Our partner has been very slow … I have no idea what is being done there and they have no idea what we do here (011).” The expectations and philosophies of the physical institution where the lab is housed took precedent over the collaborations, meaning that the institution where the simulation lab was located had greater influence in shaping the adoption and incorporation of simulation than collaborative partnerships delivering the same curricula. Two participants provided insight to this; one simulation leader stated “The barrier is … the collaboration … the two philosophies are so different … They do utilize simulation in some respect; they certainly run their simulation much differently than we (022);” while a nursing administrator added “collaborative programs … are extremely difficult to work with because … there are substantial differences just between colleges and universities in terms of how they think but I mean programs take on the personalities of their people as well (015).”

### 3.7. Shared Motivators

Collective or shared social responses or “common experiences” shared by many proved to be motivating factors of the organizational culture that shaped the adoption and incorporation of simulation. The initial reaction experienced by most institutions was one wanting to take advantage of a rare opportunity. The reaction of some programs was to apply for the funding and then, later, figure out how to use the money. One simulation leader explained “we're going to take it [the provincial simulation funding] and we'll … learn what to do with it after (010).” Wanting to take advantage of a onetime funding opportunity was a significant motivator experienced by all institutions.

What followed for most institutions were the common experiences of uncertainty and concern. Once the funding and equipment were in place, the uncertainty sets in. People were unsure of what to do with the equipment, of what the equipment was capable, and of how simulation might impact their workload and teaching responsibilities. This resulted in avoidance in many organizations, some not even opening the boxes until two to three years had passed. What helped some institutions move past the uncertainty was primarily the creation of the simulation leader role. Once this person was employed, expectations of this role developed and included learning about the potential of the equipment, how to set it up, and how to use it. While the uncertainty dissipated over time in the high-uptake sites, the low-uptake site continues to be immobilized and indecisive about simulation. This was highlighted by one faculty member there who stated “we got the equipment out of the boxes and put it in a closet; we do not use it very often and there really is no one to show us how to use it (027).”

Concern, as a shared motivating factor, was related to not wanting to “be left behind” and to sustainability. Many mid-uptake sites and some high-uptake sites that moved slower through the process of adoption and incorporation experienced uneasiness about not being as competitive as other institutions that were utilizing simulation to a greater level or with more consistency. They expressed concern over recruitment of students and felt that the slow or minimal uptake of simulation could be a deterrent for some students. Several participants also shared the belief that simulation was an expectation in all health related education programs and if they were not able to implement simulation as a teaching strategy, they would “be left behind”. The angst about being left behind is driven by the concept of time as a measurement of success. While there was neither an expected timeline nor clearly articulated time sensitive goals required in the initial proposal, many institutions measured their success in terms of time. The high-uptake sites, because of the leadership and past experiences managing this type of change, navigated the adoption more rapidly than other sites and placed a value of success on this as noted by one nursing administrator expressing pride in the short time frame her nursing program took from “conception to integration” stating “it was less than one year (012).” Time was also emphasized by a nursing faculty member at a mid-uptake sight who stated “I have shamed a few people into doing it because everybody else was doing it[simulation], so we better get going with it [simulation]” (017). On the far end of the uptake spectrum is the low-uptake site where the simulation leader expressed frustration and embarrassment stating “despite being eight years into this initiative to have it only in one course it is just kind of weird … we are so far behind in simulation (027).”

The other concern common among all mid and high-uptake sites was the issue of sustainability. Since the funding was a one-time nonsustainable resource, there was insecurity as to how to implement and sustain it once in place. There were also concerns about the equipment breaking or implementing it to a level within the curriculum where the demand would outweigh the availability, thus resulting in the need to purchase additional equipment but not having the funds. While the concern about being “left behind” has lessened for some institutions, it remains a concern for many, especially for the sites which have collaborative partners who outrival them with simulation. Concern regarding sustainability remains consistent among all institutions and is not lessening over time.

Two additional shared motivational factors were positive student experiences and institutional expectations. Nursing programs are in the business of educating student nurses. Since nursing is a practice-based profession, much learning comes from interaction with and practice of psychomotor skills on patients. The adoption and incorporation of simulation does not negate nor replace the learning from and with patients but it provides an opportunity to practice and refine skills prior to them taking place with patients. This enables students to practice in a safe and secure setting without concern for patient safety. One faculty member summed this up stating

[Students] need to demonstrate before they can do it [on patients], [simulation] allows them to experiment, make mistakes, and figure it out for themselves. Clinical teachers are out there to protect the patient so [during simulation] their mindset isn't “this isn't a patient that needs protecting but a group of students who can learn from making mistakes (008).”

Positive student responses to simulation provided the most significant motivating factor for moving forward with the implementation of simulation. Participants consistently reported on students' feelings of security working with simulated patients, relief that they could practice without harm, and that all students could benefit from the learning instead of the one lucky enough to have an experience during clinical training. Participants said it reduced anxiety and competition between students because they were all provided with the same experience. One simulation leader clarified this stating

“Instead of having only one or two students have the learning experience in the clinical setting we can set up experiences … with simulation we can make sure all of our students have experiences they may not always get (001).”

The students' responses were a profound motivating factor for participants of this study consistently among all of the mid- and high-uptake sites. Two statements by participants summed this up, one by a faculty member who stated “I think what motivates us to do [simulation] is the student's reaction (010),” and the other by a simulation leader who stated “the feedback we get is so positive from the students and the instructors … we feel like we can see the learning happening (020).” Student responses appeared to energize the participants and spur them onto further use simulation as a teaching/learning strategy.

The final motivating factor of the organizational culture stemmed from the overarching institutional expectations such as workload and the value of independence. Workload expectations are a significant driving force when adopting a new innovation, particularly one as extensive as simulation. There is a considerable amount of learning, trialing, and time required to become proficient in running the equipment, developing learning experiences, and implementing them. Some institutions have no way to accommodate this into workload assignments which results in people taking this on in their own time, aligning the work required with their own personal goals or research interests, or not participating at all. One simulation leader highlighted this saying

“It's hard to know [if] it's an expectation of the organization. It seems like if you're tenure track you would never do this kind of work load … I have … 70% teaching and they would have 40% teaching and 40% research … Their teaching work load allocation is really different because their research is way up (024).”

Due to the different workload agreements, some of the college sites were able to adjust workloads to include simulation placing them at the higher end of the uptake continuum. Independent organizational culture that holds the time-honoured practice of academic freedom above other values provides unique challenges for nursing programs wanting to adopt or integrate simulation. One nursing administrator confirmed this point stating “the university culture is such that we all have to be individuals to get ahead, to get tenure and promotion, and do research which becomes a very competitive, isolating experience (005).” A faculty member shared her view of this stating “if the expectation came from on high that thou shall implement [simulation], I can't see that happening because we could always preach academic freedom; I don't see how they could ever tell us we had to (003).” This level of independence that is the standard in many academic nursing programs creates unique challenges when trying to adopt and incorporate an innovation that requires a significant amount of extra time and a high degree of teamwork. The shared dynamic is a key organizational element that drives people to act and make decisions.

### 3.8. Scaffolding as a Way to Manage Change

This element of organizational culture included support and structure as a framework to facilitate movement through the process of adoption and incorporation. It involved reaching outside the nursing program and linking with in-house partners to share resources. This proved to be beneficial in many high-uptake sites, especially when requesting resources that benefit more than one program within the same institution. It also included the sharing of personnel resources and expertise which in turn provided an institutional cost saving strategy. Securing the framework to support simulation outside of the nursing program served to move simulation forward and to contribute to incorporation as it became a faculty wide initiative rather than just a nursing program initiative.

As this option was not available to all institutions some chose to secure support by linking with community partners to share resources and expertise. Essentially this provided the similar support as noted with the institutions that linked with their in-house partners; it made the simulation initiative larger than just the nursing program. The benefit of connecting and linking with others for support in turn raises the profile of the initiative, as summarized by one nursing faculty member who stated “a new building was built for the faculty … there was space in that new building allocated for clinical education, and simulation for all the health sciences and we also rent it out to groups like the hospital educators (014).” Sharing resources was a way to manage change, and using the same lab, equipment, or personnel proved to be beneficial. Creating links beyond the nursing program strengthened the scaffolding and contributed to the level of uptake. Institutions which chose not to link with community partners or did not have in-house partners to link with were at a disadvantage since the simulation initiative was supported primarily by just the nursing departments, and this led to a lower level of uptake.

Scaffolding to manage change intersects with all of the other organizational elements and is essential to accommodate initial and ongoing changes. It includes being creative with limited resources, providing recognition for extra work, and orienting new members.

While links external to the nursing program provided extra support and facilitated movement through the process of adoption and incorporation, there were two strategies within the nursing programs that provided support and created a structure to promote simulation as a teaching strategy. The first was to provide recognition for the extra work required. Consistently, participants stated that the work which was required to make simulation part of the curriculum was unparalleled to anything they had previously experienced. One nursing faculty member stated “we're actually supposed to do three hours a week for sim, which if you multiply by a 15 week semester, is 45 hours a semester, I do 45 hours in 3 days (010).” Institutions that compensated or recognized the extra work involved were higher on the uptake continuum than those that did not. Recognition was acknowledged in the form of education and travel that was paid for by the institution. This included sending people to conferences, workshops, or seminars. One nursing administrator discussed recognition stating “[simulation leaders] get a lot of rewards because these are the folks we are sending to conferences all over the world (015).” Recognition also came in the form of role designation, giving the simulation leaders a new and specific title. Some titles included simulationist, sim specialist, or simulation champion. What this did was to differentiate the simulation leaders and give them a new status within their institution. Recognition provided incentive for those involved in simulation, thus strengthening the support system to facilitate the incorporation of simulation.

While recognition is an astute place to start with the development of a new role, one aspect which could have strengthened the scaffolding and provided additional support would be the addition of a job description and annual appraisal documents that were reflective of the role and the work therein. Most participants stated their annual evaluation had nothing to do with their work relating to simulation. Few appraisal templates were shared with the researcher as participants voiced there was little to no connection between the work they do and their annual evaluation process. This was highlighted by one nursing administrator who stated: “the appraisal is just me reporting what I've done. There's nothing that comes out of the appraisal process that forces me to teach in a particular way (006).” One simulation leader from a mid-level uptake site interpreted this disconnection as a devaluation of her work and her teaching methods, stating “there is a lack of associated respect or merit placed on any of [simulation] activities (009).” While recognition is one strategy to strengthen support and provide a framework for success, adding specific job descriptions and having people evaluated for the work they do are strategies that could be used to add an additional level of structure and support to a newly developing role in nursing.

The last scaffolding strategy that was consistent among the majority of the high-uptake sites was the orientation of new members. Orientation of current and new nursing faculty members and staff is a strategy to maintain change and assign value to the innovation. One nursing administrator stated “we focused on everybody getting a baseline understanding of simulation … so new faculty and staff goes to the [simulation leader] for training (012).” Other examples of orienting new members included training modules for new faculty and the availability of individual training. Orientation was a consideration in both of the rapid high and a couple of slower high-uptake sites, but most of the other sites had not given this much consideration as stated by one faculty member “we don't really have a process for [orientation] (023).” Using strategies such as being creative with resources, providing recognition for extra work, and orienting new members proved to be effective scaffolding strategies that contributed to the adoption and incorporation of simulation.

## 4. Discussion

The organizational culture of nursing programs and the academic institutions in which they are situated contribute significantly toward the adoption and incorporation of simulation into the curriculum. This study revealed that five key organizational elements contribute to the adoption and incorporation of simulation into nursing curricula. The OESSN model shows these organizational elements as bordered by the institutional, faculty, or nursing program guiding philosophies. The connection between the beliefs and values and the leaders in the OESSN was portrayed as a two-way path. This dual direction concept adds to Schein's [26] theory by suggesting that movement between and among these concepts exists and can facilitate the uptake of an innovation into an academic program. Schein purports that beliefs and values impact actions and decisions, but this study reveals that movement from the beliefs and values is not unidirectional; flowing downward, the guiding philosophies influence the decision making of leaders; flowing upward, the leaders used the values and beliefs as rationale to support their requests for additional resources. Organizational philosophies are an important aspect that should be used to guide the adoption and incorporation of simulation. Despite funding constraints in academia, a recommendation resulting from this study is that when requesting funding support, nursing programs strategically use the guiding philosophies as tools to negotiate the needed resources when adopting or incorporating an innovation.

An important consideration from the findings of this research is the shared emotional reaction to time as a driving force. The initial emotion of not wanting to be left behind and the ongoing emotion related to perceptions of success both denote time by the form of measurement. This concept aligns with theories noted in social sciences. Graham [28] suggests that time is a concept of culture that influences people's perceptions. He further suggests that the completion of tasks is directly related to time, and a task that is not completed or takes additional unanticipated time is often referred to as “being behind” (page 335). Graham further suggests that time spent completing tasks positions people or institutions for future progress. This is consistent with what was found in this study when participants expressed feelings of satisfaction with how far they had progressed with the adoption and incorporation of simulation over a short period of time. This finding is both surprising and predictable. Surprising because few institutions were able to articulate goals related to simulation therefore making it difficult to measure accurately their success or validate their progress or lack thereof. The concept of time as a measurement of success is simultaneously predictable within the context of this study. Researchers have suggested that North America typically has a monochronic perception of time, where the focus is future oriented and concerned with completing tasks within set time frames [28]. There is also the philosophy that time can be wasted and that time provides meaning and affects judgment [29, 30]. The result of measuring progression and or success by time when there is no clear vision or goals related to the adoption and intergradations of simulation causes unnecessary emotional reactions which can become a barrier. One recommendation is to take lessons from polychronic time oriented cultures that focus on participation and achievement of milestones rather than fixed or unreal time frames [30]. Many aspects of adopting and incorporating an innovation are multifaceted and require many levels of cooperation within the organizations that takes time. Recommendations from this study would be to set clear measurable goals related to the adoption and incorporation of simulation and the purpose that it will serve in the program. The quality and achievement of goals should then be evaluated directly rather than using time as a measurement of success. This research provides insight into the emotional and cultural concepts of time when adopting and incorporating simulation and could generate future research in this area.

Using Schein's [26] theory sensitized the researchers to the concept of space as an organizational element. While space as a concept has the potential to be interpreted in a variety of ways, the findings of this research suggest that the physical locale is an essential driving force within the context of adopting and incorporating simulation into nursing curricula. While space is a commodity at many postsecondary institutions [31], this study found it was more than just the availability of space; the more important facet was the location of the space. Space is a value laden resource. The physical location of the lab elicited emotional responses such as isolation or questions of value. Nursing program members perceived simulation to be undervalued or not valued if the allocated space was not ideally located nor within close proximity to the nursing department. Recommendations would be to encourage leaders to negotiate desired space close to the department if possible. If not, then leaders should communicate that space in academic settings is a scarce resource, a commodity, and that value exists even when the space is less than ideal.

Schein [15] states that the orientation of new members can perpetuate the values and beliefs of an organization. This was noted in a few of the high-uptake sites in this study but, overall, was limited in the other sites. Schein suggests that knowledge transferred to new members reveals what the institution values. He further states that the culture of the organization is taught to new members so that they can learn how to think, feel, and act in relation to the work environment. Not including simulation as part of the orientation of new members may lead to its devaluation, or new hires might view it as optional. Recommendations would be to include simulation in the orientation of new members. This provides an opportunity to show the value of simulation as a teaching strategy and perpetuates it as part of the culture.

The findings from this study indicate that there are many motivating factors involved at the level of the nursing program including the positive responses of students toward simulation as a teaching strategy. While there is not a substantial body of evidence supporting the effectiveness of simulation to positively influence the acquisition of new or sustained clinical skills [32, 33], organizations are highly motivated and focused on implementing simulation for the outcome of improved student satisfaction and student engagement with the learning environment.

The connections and the fluidity between and among the key organizational elements were an important finding from this study. The five organizational elements link together and are interdependent. Having this understanding of the interconnectedness of the organizational factors provides an opportunity for leaders to facilitate change. For example, the institutional expectations link directly to scaffolding. It is an expectation in most institutions that some form of annual appraisal is done. Participants in this study shared that they felt their work related to simulation was not reflected in this process. This disconnection provides an ideal opportunity for leaders to communicate, share information, and align work contributions so they fit within the parameters of the appraisal process. This is only one example of the interconnectedness of the key organizational elements. Other than the inherent link between leadership and communication [15, 34], the concept of interconnectedness adds to the literature on organizational culture. The dimensions of organizational culture are often presented in isolation of each other [15, 34, 35]. Having an awareness of this would provide an advantage to nursing programs wanting to incorporate simulation because they could look at these aspects from a holistic viewpoint, knowing that one element of organizational culture impacts the other. Being cognizant of this when creating strategies to enhance this synergy could facilitate the adoption and incorporation of simulation as well as other innovations into nursing curricula.

### 4.1. Strengths and Limitations

Multiple strategies were used to enhance rigour in this study. Member checking was achieved during second interviews [19]. Triangulation of data sources and data types enhanced quality and credibility. The inclusion of multiple sites added to the transferability of the findings [36]. An audit trail consisting of memos, reflexive journaling, and field notes was maintained throughout the study to maintain confirmability [37, 38].

This study did not include an exploration of nursing program, faculty, or institutional finances, flow of funding within organizations or among collaborative programs, nor union agreements. Inclusion of these aspects may have elicited additional findings related to the organizational elements that shape the adoption and incorporation of simulation into nursing curricula.

## 5. Conclusions

The organizational cultures of nursing programs in Ontario that shape the adoption and incorporation of simulation into curricula has been represented by the OESSN model that depicts the five key organizational elements: leaders, information exchange, physical locale, shared motivators, and scaffolding as a strategy to manage change. This paper was conceptualized from interviews with nursing administrators, nursing faculty members, and simulation leaders as well as a review of key documents from 13 different nursing programs. This research has contributed to the literature on simulation and enhanced the literature on organizational culture. The OESSN model provides a framework that nursing programs could use to initiate or further facilitate the adoption and incorporation of simulation into curricula. It provides insight into key organizational elements that should be considered when incorporating simulation.

## Figures and Tables

**Table 1:** Demographic information.

Age	
20–30	0
31–40	8.3%
41–50	37.5%
51–60	37.5%
61–70	16.6%
Primary place of employment	
College	44.4%
University	55.5%
Years of experience using mid- to high- level simulators	
No experience	12.5%
1–3 years	16.6%
3–5 years	37.5%
5–10 years	25%
10–15 years	4.1%
20 plus	4.1%

**Table 2:** Documents reviewed.

Level within the institution	Type of documents	Total reviewed	Publicly available	Provided
Institutional	Institutional philosophy Institutional mission statement Institutional vision statement Institutional values statement Institutional core values statement	24	24	—

Faculty	Faculty philosophy Faculty mission statement Faculty vision statement Staff performance evaluation Performance appraisal Faculty annual appraisal template Annual performance evaluation report Support staff performance appraisal	9	5	4

Nursing program	Nursing program philosophy Nursing program mission statement Nursing program vision Nursing program learning outcomes	24	23	1

Simulation specific	The original simulation proposal Simulation centre strategic plan Simulation development template Sim center mission statement Sim centre vision statement Simulation strategic plan Strategic plan for simulation in nursing Application to nursing secretariat for simulation funding Simulation centre strategic plan	10	—	10
